# Endocrinological analysis of endothelium-dependent vasodilation in middle-aged patients with impaired glucose tolerance during prediabetes mellitus

**DOI:** 10.3892/etm.2014.1482

**Published:** 2014-01-09

**Authors:** YIPING LIU, JIANWEI LI, ZHENGHONG ZHANG, YEDONG TANG, ZUOSONG CHEN, ZHENGCHAO WANG

**Affiliations:** 1 Laboratory of Sport Physiology and Biomedicine, School of Physical Education and Sport Sciences, College of Life Sciences, Fujian Normal University, Fuzhou, Fuijian 350007, P.R. China; 2 Provincial Key Laboratory for Developmental Biology and Neurosciences, College of Life Sciences, Fujian Normal University, Fuzhou, Fuijian 350007, P.R. China; 3 Department of Ultrasound, Fujian Provincial Hospital, Provincial Clinical College of Fujian Medical University, Fuzhou, Fujian 350001, P.R. China

**Keywords:** endothelium-dependent vasodilation, impaired glucose tolerance, diabetes mellitus, cardiovascular disease

## Abstract

Impaired glucose tolerance (IGT) is a pre-diabetic metabolic state involving heterogeneous and dynamic changes between the normal and diabetic state. The present study aimed to investigate the endocrine regulation of endothelium-dependent dysfunction in middle-aged patients with IGT and in patients with a normal glucose tolerance (NGT). An oral glucose tolerance test was performed to determine the NGT and IGT states. Physiological and biochemical analyses were performed. The carotid artery structure and function were investigated with Doppler supersonic diagnostic equipment. The functioning of the vascular endothelium was analyzed with physiological and biochemical indices in the IGT group. The results showed a significant reduction in endothelium-dependent vasodilation, but not in endothelium-independent vasodilation in the IGT group compared with those of the NGT group. It was identified that the intima-media thickness of the carotid artery and expression levels of endothelin-1 were significantly higher, whereas the endothelium-derived factor C-type natriuretic peptide levels were significantly lower in the IGT group compared with those in the NGT group. Notably, significant correlations were identified between endocrinological changes and body composition, including fat and glucose metabolism, in the IGT group. Our data indicate that vascular endothelial functions may be impaired by fat and glucose metabolism and body composition in IGT patients during prediabetes mellitusare.

## Introduction

Impaired glucose tolerance (IGT) is a particular metabolic state involving heterogeneous and dynamic changes between the diabetic and normal state. One of the early characteristics of prediabetes mellitus is elevated postprandial glucose levels. (1,2) During the IGT stage, insulin resistance (IR) and disorders of glucose metabolism commonly occur (3). IR is strongly associated with hyperinsulinemia, which results in the excessive secretion of insulin for maintaining normal physiological functions (3). Moreover, IR often occurs along with IR of hepatic and muscle cells, increases of glycogen production and output, reduction of insulin-stimulated glucose uptake and its manifestations, postprandial hyperglycemia, and reductions of dystrophin-glycoprotein generation and storage, which results in a weakening of the suppressive effect of insulin on lipolysis (2,4,5). At present, IGT and diabetes mellitus (DM) with IR have become increasingly common due to the prevalence of unhealthy lifestyles (6–8).

IGT has been a subject of study for many years. In the 1980s, the Chinese Daqing test increased the understanding of diabetes and IGT prevention (9). Subsequently, several diabetes prevention studies, such as a diabetes prevention study conducted in Finland (10) and a diabetes prevention program in the USA (11), showed that the incidence of diabetes is reduced significantly following lifestyle intervention during the IGT phase. Therefore, IGT is a crucial stage for the prevention of DM.

The risk of cardiovascular disease (CVD) in patients with IGT is similar to that in patients with DM (12,13); therefore, IGT may not only result in diabetes, but also increase the risk of CVD (13). A key prognostic indicator for CVD is endothelial dysfunction, which is responsible for vasodilation (14). Strongly correlated with obesity and IR, endothelial dysfunction may result in vascular complications of DM and be important in CVD pathogenesis (3,13,14). Thus, the treatment of endothelial dysfunction via early-treated IGT intervention has become increasingly important for the prevention of the cardiovascular complications of DM.

Given the complex endocrinological changes of endothelium-dependent vasodilation in patients with IGT during prediabetes mellitus, the present study investigated the physiological and biochemical indices of glucose and fatty acid metabolism and body composition in normal glucose tolerance (NGT) and IGT groups. Regression and correlation analysis was performed to investigate vascular endothelial functions in patients with IGT with the aim of identifying an effective method for preventing DM and CVD in the early stages.

## Materials and methods

### Study subjects and groups

In this study, 61 patients with IGT (aged, 49.8±4.8 years; 7.8–11.1 mmol/l OGTT2h) were selected from 129 individuals who were at a high-risk of DM according to the 2004 guidelines of China diabetes prevention and control (15). Patients were selected from two residential communities (the Cangshan and Gushan districts of Fuzhou, the capital city of Fujian province, China). Twenty-two patients with NGT were chosen as the controls. The procedures of the present study were approved by the Institutional Research Ethics Board of Fujian Normal University and Fujian Medical University (Fuzhou, China). All participants of this study understood the experimental procedures and provided written informed consent.

### Oral glucose tolerance test (OGTT)

The OGTT was performed for the diagnosis of NGT and IGT according to the 2004 guidelines of China diabetes prevention and control. Diagnosis was based on the OGTT 2 h following the oral administration of 75 g glucose (OGTT2h) (16). Briefly, all participants underwent 10–16 h of fasting overnight and the OGTT commenced at ~8:00–9:00 a.m. following a 30 min rest at 20–25ºC. Following the extraction of blood samples (5 ml) from patients in a fasted state, the patients orally received 75 g of anhydrous glucose in 250–300 ml warm water over 3–5 min. Second blood samples were collected 2 h following the sugar intake.

### Measurement of morphological parameters

Measurements of morphological parameters, including BMI and body fat percentage, were made using a body composition resistance measurement instrument (Biospace InBody 3.0; Biospace Co., Ltd. Seoul, South Korea), according to the national physiological constitution monitoring requirements (15).

### Detection of physiological and biochemical indices

The biochemical indices were detected using various kits. Briefly, the blood sugar concentrations were measured immediately following the collection of blood. The biochemical indices, including C-type natriuretic peptide (CNP), endothelin-1 (ET-1), insulin, leptin, resistin, adiponectin, tumor necrosis factor-α (TNF-α) and free fatty acid levels, were detected by radioimmunoassay (RIA). Blood sugar concentrations were analyzed by the glucose oxidase method using a fully automatic biochemical analyzer (Hitachi 7020; Hitachi, Tokyo, Japan). The insulin RIA kit [intra-assay coefficient of variation (cv) <4.30% and inter-assay cv <7.10%], leptin RIA kit (intra-assay cv <10.00% and inter-assay cv <15.00%) and TNF-α RIA kit (intra-assay cv <5.00% and inter-assay cv <8.00%) were purchased from Atomic Gaoke Co., Ltd., Department of Isotope, China Institute of Atomic Energy (Beijing, China). The resistin RIA kit (intra-assay cv <3.59% and inter-assay cv <9.25%) and the adiponectin RIA kit (intra-assay cv <3.59% and inter-assay cv <9.25%) were purchased from Linco Research Inc. (St. Louis, MO, USA). The ET-1 RIA kit (intra-assay cv <10.00% and inter-assay cv <15.00%) and CNP RIA kit (intra-assay cv <10.00% and inter-assay cv < 5.00%) were purchased from the General Hospital of PLA from the Institute of Science and Technology Development Center (Haidian, China). The free fatty acid enzyme-linked immunosorbent assay kit was provided by Randox Laboratories Ltd., (Antrim, UK). All measurements were performed in strict accordance with the kit instructions. Homeostasis model assessment-insulin resistance (HOMA-IR) was calculated with the following formula: HOMA-IR = fasting plasma insulin concentrations (mIU/l) × fasting blood glucose (mmol/l)/22.5 (17).

### Supersonic evaluation of the carotid artery

Evaluation of the carotid artery structure and function was conducted using the Celermajer method (17,18). Briefly, endothelium-dependent vasodilation of the brachial artery was measured using color Doppler supersonic diagnostic equipment (iU-22; Phillips, Amsterdam, The Netherlands) with a L17–5 MHz transducer. Endothelium-independent vasodilation was calculated using the following equation: ΔDia-N (%) = (artery diameter with 0.4 mg sublingual nitroglycerin 1 min - basic diameter)/basic diameter ×100. Brachial artery endothelium-dependent vasodilation was calculated as follows: ΔDia-P (%) = (maximum diameter inside brachial artery with 1 min decompression - basic diameter)/basic diameter ×100 (17).

### Statistical analysis

All experimental data are expressed as the mean ± standard deviation. Paired t-test was used to evaluate statistically significant differences. SPSS software, version 11.5 (SPSS, Inc., Chicago, IL, USA) was used for Pearson correlation analysis and regression analysis of vascular endothelium functions with multivariate step-by-step regression analysis. P<0.05 was considered to indicate a statistically significant difference and P<0.01 was considered to indicate a marked statistically significant difference.

## Results

### Vascular endothelium functions are impaired in patients with IGT

To determine whether vascular endothelial functions were impaired in the IGT group, physiological and biochemical indices were analyzed. The CNP and ET-1 concentrations were detected. The carotid artery intima-media thickness (IMT) and basic diameter of the carotid artery were measured. Endothelium-dependent vasodilation (ΔDia-P) and endothelium-independent vasodilation (ΔDia-N) were calculated in the NGT and IGT groups. The results showed a significant difference in endothelium-dependent vasodilation, but not in the endothelium-independent vasodilation between the NGT and IGT groups (Table I). The increase in the carotid artery IMT in the IGT group confirmed our speculation regarding the effects of IGT on endothelium-dependent vasodilation. To further understand the changes of vasodilation that may result from endothelial dysfunction, the endothelium-derived factors CNP and ET-1 were tested, and the basic diameter of the carotid artery was measured. CNP and ET-1 were identified to be significantly impacted in the IGT group (Table I), indicating endothelium dysfunction. These results suggest that vascular endothelial functions are impaired in patients with IGT.

### Adipocytokines, fat and glucose metabolism and body composition are impacted in patients with IGT

To investigate the molecular mechanisms involved in the changes of endothelium-dependent vasodilation in patients with IGT, fat and glucose metabolism were also measured in the NGT and IGT groups. The results showed significantly higher expression levels of leptin, resistin and free fatty acids in the IGT group compared with those in the NGT group, and a reduction in the adiponectin level in the IGT group compared with that of the NGT group (Table I), implying the possible involvement of these fat metabolism factors in vascular endothelial function. Concentrations of TNF-α did not show a significant difference between the two groups (Table I).

Analysis of HOMA-IR and glucose metabolism showed that there were also significant differences in fasting blood glucose, OGTT2h, fasting insulin and HOMA-IR levels between the NGT and IGT groups (Table I). Analysis of body composition in the NGT and IGT groups also showed significant differences in BMI, waist circumference, waist to hip ratio and body fat percentage (Table I). These results suggest that adipocytokines, fat and glucose metabolism and body composition were abnormal in the IGT group compared with those in the NGT group.

### Adipocytokines, fat and glucose metabolism and body composition are associated with vascular endothelial functions

To further investigate the factors affecting vascular endothelial functions in patients with IGT, correlation analysis of adipocytokines and fat metabolism was conducted. This showed a significant correlation of CNP with leptin and resistin levels (Table II), and a significant correlation between ET-1 and adiponectin concentrations (Table II) in the IGT group, demonstrating that fat metabolism factors may also affect vascular endothelial functions in IGT patients. Furthermore, Pearson regression analysis was performed and the results indicated that adiponectin and OGTT2h were the major factors in the CNP regression equation (Table III). CNP regression equation was as follows: Y_CNP_ = 3.856 × X_adiponectin_ − 2.273 × X_OGTT2h_ + 2.263.

Correlation analysis of HOMA-IR and glucose metabolism showed that there was a significant correlation between ET-1 and fasting insulin, OGTT2h and HOMA-IR (Table II), and carotid artery IMT was significantly correlated with HOMA-IR in the IGT group (Table II). The results of the Pearson regression analysis of ΔDia-P showed that OGTT2h and adiponectin were the major factors affecting ΔDia-P in the IGT group (Table III). ΔDia-P regression equation was as follows: Y_ΔDia-P_ = 0.981 × X_OGTT2h_ – 1.036 × X_adiponectin_ + 7 .608

The results of the correlation analysis also showed that there was a significant correlation of body fat percentage with ET-1 and carotid artery IMT in the IGT group (Table II). The results of the Pearson regression analysis of ET-1 showed that OGTT2h, fasting insulin levels and waist circumference were involved in the ET-1 regression equation (Table III). ET-1 regression equation was as follows: Y_ET-1_ = 26.617 × X_OGTT2h_ +2.273 × X_fasting insulin_ − 2.562 × X_waist circumference_ + 65.661.

Pearson regression analysis of carotid artery IMT indicated that body fat percentage was the major factor affecting carotid artery IMT in the IGT group (Table III) and was calculated as follows: Y_Carotid artery IMT_ = 2.059 × X_body fat percentage_ + 2.059. The above results suggest that vascular endothelial functions are affected by adipocytokines, glucose and fat metabolism and body composition.

## Discussion

This study clearly showed that endothelium-dependent dysfunction is an important early pathophysiological change in patients with IGT during prediabetes mellitus and that endocrinological changes may also be involved in the regulation of this process.

Since the 1980s, studies have focused on the prevention of DM in patients with IGT (9,12,13). Patients with IGT are in a sub-healthy condition, which may not only result in DM, but also increase the risk of CVD (9,12,13). Dysfunction of the vascular endothelium is closely associated with CVD (3,13) and DM complications (14). Since patients with IGT have few clinical symptoms and are not frequently analyzed, the present study combined outpatient records and physical examination records of local residents of two communities, the Cangshan and Gushan districts, to achieve a higher cohort of patients with IGT. The results showed significant impacts of IGT on endothelium-dependent vasodilation in middle-aged patients based on the results of ΔDia-P; however, IGT did not significantly impact ΔDia-N. The increase of carotid artery IMT in the IGT group compared with that in the NGT group was consistent with this.

ET-1 is a small vasoactive polypeptide, which has the strongest effect *in vivo* and the longest duration of action of known vasoconstrictive substances; its vasoconstrictive effects are mediated through binding with the endothelin receptor (19,20). CNP is also a vasoactive peptide, which inhibits the proliferation and migration of vascular smooth muscle cells and formation of the extracellular matrix as well as inducing vasodilatation (10,20). Results of the present study showed that bioactive factors in the IGT group differed from those in the NGT group, indicating endothelial pathogenesis in the IGT group.

The current study further identified significant differences in the levels of leptin, resistin, adiponectin and free fatty acids in the IGT group from those in the NGT group. The results of regression and correlation analyses indicated their roles in the secretion of endothelial factors and vasodilation, similar to glucose metabolic factors, which implied possible abnormal endocrinological regulation in patients with IGT during prediabetes mellitus. The analysis of the body composition index suggested its putative role in vascular endothelial function.

To the best of our knowledge, the present study is the first to provide detailed clinical data and regression analysis equations of vascular endothelial functions in patients with IGT during prediabetes mellitus. This may aid further studies of patients with IGT and may lead to the identification of novel treatments for preventing DM and CVD during the early stages.

## Figures and Tables

**Table I:** Comparison of physiological and biochemical indices between the NGT and IGT groups.

Physiological and biochemical indices	NGT (n=22)	IGT (n=61)	t-value	P-value
Vascular endothelium functions				
CNP (pg/ml)	24.720±8.281	14.702±4.544	7.004	<0.01**
ET-1 (pg/ml)	116.246±15.887	143.797±34.465	3.603	<0.01**
Carotid artery IMT (mm)	0.894±0.171	1.019±0.194	2.359	<0.05*
Basic diameter of carotid artery (mm)	4.366±0.709	4.445±0.675	0.465	>0.05
ΔDia-P (%)	16.670±6.259	11.727±3.571	4.388	<0.01**
ΔDia-N (%)	18.873±6.555	17.396±3.730	1.268	>0.05
Adipocytokines and fat metabolism				
Leptin (ng/ml)	10.078±3.968	17.334±4.401	12.108	<0.01**
Resistin (ng/ml)	19.541±5.132	23.912±5.220	3.452	<0.01**
Adiponectin (mg/l)	11.197±4.720	8.736±3.494	8.166	<0.05*
TNF-α (mg/)	1.379±0.306	1.589±0.473	1.933	>0.05
Free fatty acid (mmol/l)	0.579±0.192	0.763±0.203	6.166	<0.01**
HOMA-IR and glucose metabolism				
Fasting blood glucose (mmol/l)	5.086±0.378	5.733±0.4797	2.434	<0.05*
OGTT2h (mmol/l)	6.406±0.640	9.348±0.580	19.843	<0.01**
Fasting insulin (mIU/l)	9.460±2.460	26.902±7.835	10.226	<0.01**
HOMA-IR	2.451±1.707	6.965±2.932	9.290	<0.01**
Body composition				
BMI (kg/m^2^)	24.311±1.732	27.249±2.194	10.062	<0.01**
Waist circumference (cm)	83.926±5.710	91.473±4.638	8.692	<0.01**
Waist to hip ratio	0.850±0.082	0.898±0.096	2.392	<0.05*
Body fat (%)	20.566±3.929	25.469±2.803	9.998	<0.01**

NGT, normal glucose tolerance; IGT, impaired glucose tolerance; CNP, C-type natriuretic peptide; ET-1, endothelin-1; IMT, intima-media thickness; ΔDia-P, endothelium-dependent vasodilation; ΔDia-N, endothelium-independent vasodilation; TNF-α, tumor necrosis factor-α; HOMA-IR, homeostasis model assessment-insulin resistance; OGTT, oral glucose tolerance test; BMI, body mass index.

* P<0.05 and

** P<0.01.

**Table II:** Correlation analysis of vascular endothelium functions in the IGT group.

Correlation analysis	CNP (pg/ml)	ET-1 (pg/ml)	Carotid artery IMT (mm)	ΔDia-P (%)
HOMA-IR and glucose metabolism				
Fasting insulin (mIU/l)	−0.046	0.452**	−0.239	0.030
Fasting blood glucose (mmol/l)	−0.227	0.202	−0.230	0.159
OGTT2h (mmol/l)	−0.170	0.497**	−0.189	0.269*
HOMA-IR	−0.075	0.411**	−0.285*	0.014
Body composition				
BMI (kg/m^2^)	−0.023	0.230	−0.091	0.058
Body fat percentage (%)	0.060	0.409**	−0.332**	0.047
Waist circumference (cm)	−0.013	0.152	−0.136	0.110
Waist to hip ratio	−0.116	0.212	−0.182	0.132
Adipocytokines and fat metabolism				
Leptin (ng/ml)	0.392**	−0.140	0.012	−0.082
Resistin (ng/ml)	0.288*	−0.077	0.228	0.134
Adiponectin (mg/l)	0.214	0.336**	−0.222	−0.206
Free fatty acid (mmol/l)	0.215	−0.076	0.106	0.062

IGT, impaired glucose tolerance; CNP, C-type natriuretic peptide; ET-1, endothelin-1; IMT, intima-media thickness; ΔDia-P, endothelium-dependent vasodilation; HOMA-IR, homeostasis model assessment-insulin resistance; OGTT, oral glucose tolerance test; BMI, body mass index.

* P<0.05 and

** P<0.01.

**Table III:** Pearson regression analysis of vascular endothelium function items.

Pearson regression analysis	B	SE	β	t-value	P-value
CNP					
Constant	2.263	11.358		0.199	0.843
Adiponectin	3.856	1.135	0.419	3.397	0.001**
OGTT2h	−2.273	0.966	−0.290	−2.352	0.022*
ET-1					
Constant	65.661	89.596		0.733	0.467
OGTT2h	26.617	6.631	0.448	4.014	<0.001**
Fasting insulin	2.273	0.590	0.517	3.854	<0.000**
Waist circumference	−2.562	1.008	−0.345	−2.541	0.014*
Carotid artery IMT					
Constant	2.059	0.338		6.092	<0.000**
Body fat percentage	−0.036	0.013	−0.332	−2.701	0.009**
ΔDia-P					
Constant	7.608	3.975		1.914	0.061
OGTT2h	0.981	0.338	0.362	2.901	0.005**
Adiponectin	−1.036	0.397	−0.326	−2.608	0.012*

CNP, C-type natriuretic peptide; OGTT, oral glucose tolerance test; ET-1, endothelin-1; IMT, intima-media thickness; ΔDia-P, endothelium-dependent vasodilation; B, regression coefficient; SE, standard error; β, standardized coefficient.

* P<0.05 and

** P<0.01.
